# *Haemophilus influenzae* Type b Meningitis in the Short Period after Vaccination: A Reminder of the Phenomenon of Apparent Vaccine Failure

**DOI:** 10.1155/2012/950107

**Published:** 2012-08-16

**Authors:** Noa Greenberg-Kushnir, Orly Haskin, Havatzelet Yarden-Bilavsky, Jacob Amir, Efraim Bilavsky

**Affiliations:** ^1^Department of Pediatrics C, Schneider Children's Medical Center, 14 Kaplan Street, 49202 Petach Tikva, Israel; ^2^Sackler Faculty of Medicine, Tel Aviv University, 69978 Tel Aviv, Israel; ^3^Department of Pediatrics A, Schneider Children's Medical Center, 14 Kaplan Street, 49202 Petach Tikva, Israel

## Abstract

We present two cases of bacterial meningitis caused by *Haemophilus influenzae* type b (Hib) which developed a few days after conjugate Hib vaccination. This phenomenon of postimmunization provocative time period is reviewed and discussed. These cases serve as a reminder to clinicians of the risk, albeit rare, of invasive Hib disease in the short period after successful immunization.

## 1. Introduction

*Haemophilus influenzae* type b (Hib) was the leading cause of bacterial meningitis in children worldwide until the introduction of the Hib conjugate vaccine in the early 1990s [1]. Since then, the incidence of Hib disease has declined dramatically in high-income countries and virtually eliminated in parts of the United States and Europe [1].

In 1994, the Hib-conjugated vaccine was introduced into the Israeli National Immunization Program. In 1997, a four-dose vaccine schedule was adopted, given at 2, 4, 6, and 12 months of age. Prospective surveillance estimated that vaccine effectiveness was 95% (95% CI 92–96%) against any invasive disease and 97% (95% CI 93–98%) against bacterial meningitis [2].

Nevertheless, over the past 20 years, there have been some reports of invasive Hib disease within a short period after administration of the vaccine [3–5]. This report describes two children in whom Hib meningitis developed a few days after vaccination. These cases serve as a reminder for clinicians of a phenomenon of elevated risk for infection and apparent vaccine failure in the short period after Hib immunization.

## 2. Case Reports

### 2.1. Case 1

A 10-week-old girl presented to another hospital with fever, refusal to eat, grunting respirations, and hypertonicity of 48-hour duration. All symptoms began one day after she had received the first dose of the combination Infanrix-IPV+Hib vaccine (a combined vaccine against diphtheria, tetanus, pertussis, polio, and Hib infections). Her parents reported that she had been perfectly healthy the day before vaccination. 

Past medical history revealed that the patient had been born at 31 weeks' gestation after premature rupture of the membranes; maternal fever was documented during delivery. She was hospitalized in the neonatal intensive care unit and treated with empiric antibiotics for 3 days pending blood culture results. The rest of her hospitalization was uneventful, and she was discharged at the age of 5 weeks in good medical condition. 

At the present admission to the other hospital, bacterial meningitis was suspected on the basis of abnormal cerebrospinal fluid (CSF) cell count (2358/mm^3^, with neutrophil predominance 60%), protein, and glucose (235 mg/dL, 1 mg/dL, resp.) despite negative findings on direct microscopy of a CSF sample. Empiric treatment with ceftriaxone, vancomycin, and dexamethasone was started. Two days later, both blood and CSF cultures grew *Haemophilus influenzae*, which was identified as type b using latex agglutination-based antigen detection test. The patient's clinical status gradually improved over the next 4 days, when a secondary fever was noted in addition to new-onset seizures. Treatment with phenobarbital was initiated, and the patient was transferred to our tertiary medical center.

At admission to our department, magnetic resonance imaging (MRI) study revealed subdural fluid collections in the posterior fossa and around the hemispheres. Given the patient's clinical and neurological deterioration as well as the high levels of inflammatory markers, a tentative diagnosis of subdural empyema was made. The patient was transferred to the neurosurgery department where she underwent bilateral craniotomy. Findings included a subdural empyema with severe brain edema. The empyema was drained. The antibiotic treatment was continued and combined with anticonvulsant and supportive treatment, leading to gradual improvement.

The patient was discharged from our institute after 20 days, during which she received ceftriaxone. On her discharge, she was clinically stable and had normal findings on neurologic examination except for mild hypertonicity of the left arm and mild left torticollis. On follow-up visits, 2 months later and at age 1 year, brainstem-evoked response audiometry (BERA) was within normal range. There was a mild global developmental delay with normal findings on neurologic examination.

### 2.2. Case 2

A 5-month-old boy presented to our hospital with fever, apathy, vomiting, and diarrhea of 24-hour duration. All symptoms began 6 days after he received the second dose of the Infanrix-IPV+Hib vaccine. His parents reported that he had been perfectly healthy on the day before vaccination. 

 Past medical history was unremarkable. The patient was born after a normal term pregnancy and vaginal delivery. He received the first dose of Infanrix-IPV+Hib vaccine at age of 2 months without adverse events.

At admission, the patient was febrile and apathetic, with grunting respiration and a bulging fontanel. Lumbar puncture revealed a white blood cell count of 4,000 cells/mm^3^, 95% segmented neutrophils, and glucose level of 8.4 mg/dL (protein level was not calculated because of technical problem). Gram staining of the CSF was negative. Empiric treatment with ceftriaxone, vancomycin, and dexamethasone was started. After 36 hours, blood culture grew *Haemophilus influenzae*, which was later identified as type b using latex agglutination-based antigen detection test. 

Over the next days, the patient continued treatment with ceftriaxone, with gradual improvement. BERA study was normal. He was discharged home after 11 days in excellent condition, with no neurologic deficits.

## 3. Discussion

The Hib vaccine targets the organism's capsular polysaccharide, polyribosylribitol phosphate (PRP). To increase immunogenicity and induce immune memory, several conjugate vaccines were developed through covalent linkage of PRP to a carrier protein. Four conjugated vaccines were found safe and were introduced into routine immunization programs worldwide [1]. 

While the introduction of conjugate vaccine against Hib has had a substantial impact on Hib infection, over the past 20 years, sparse reports of cases of invasive disease after Hib vaccination have been published [3–5]. Booy et al. [3] investigated all cases of invasive Hib infection that occurred over a 3-year period in children in the United Kingdom after they received at least one dose of the Hib-conjugate vaccine. They identified two kinds of vaccine failures: apparent (early) and true (late). True failures were defined as Hib invasive disease occurring either >1 week after a child up to the age of 12 months received at least two doses of the vaccine, or >2 weeks after a single dose was received by a child >12 months of age. Hib invasive infections that occurred within one week after the administration of one or two doses of vaccine were considered apparent vaccine failures. Thus, in the present report, both cases represent apparent (early) vaccine failures.

The “apparent vaccine failure" was a known phenomenon of the early polysaccharide vaccine [6], but relatively rare when attributed to conjugate vaccine. In Booy's work [3], they reported of 46 apparent vaccine failures out of the 164 cases of invasive disease among the entire population of United Kingdom vaccinated children. Singleton et al. reviewed data from Alaska's Statewide Disease Surveillance conducted during 1980–2004 [4]. Study population included 103,000 children younger than 10 years of age. They reported of 3 early vaccine failures out of 44 cases of invasive disease in immunized children. Cowgill et al. reviewed hospitalization data of a main district hospital in Kenya and reported of 24 cases of invasive disease in immunized children, 12 of them early failures [5]. 

Already in 1901, Wright [7] coined the term “negative phase" to describe the decrease in bactericidal activity; he observed 1 to 21 days after administration of typhoid vaccine. This phenomenon of postimmunization provocative disease was also confirmed in early studies of conjugated and unconjugated Hib vaccines which reported that subjects with preexisting anticapsular antibodies showed a decrease in antibody concentrations after immunization [8, 9]. The nadir in antibody decline was reached 2-3 days after immunization, and concentrations normalized by day 7. The magnitude of the decline was negatively correlated with the preimmunization concentration [9]. This decrease is presumed to occur with all 4 available Hib conjugate vaccines [9]. Some authors attributed these findings to the formation of a complex between the vaccine antigens and the preexisting serum antibodies, which induces a transient decline in antibody concentration [10]. This could pose a risk of invasive disease if it occurs during a period of asymptomatic colonization with Hib [10].

In order to understand whether the individual having received the Hib vaccine is adequately protected against the organism, the level of anti-PRP antibodies should be assessed. The exact mechanism underlying the invasive infection in our patients could not be determined because the concentration of Hib antibodies was not measured in either case before or after immunization. However, these cases are reported to serve as a reminder to clinicians of the risk, albeit rare, of invasive Hib disease in the short period after successful immunization. Clinicians should bear this possibility in mind when starting empiric antibiotic treatment in children who present with signs of infection within a week of receiving the vaccine. Large-scale studies that focus on this time frame are still needed.
